# Extreme resistance to thyroid hormone caused by a novel mosaic thyroid hormone receptor beta mutation

**DOI:** 10.1530/ETJ-25-0092

**Published:** 2025-11-11

**Authors:** Ferdy S van Geest, Wenjun Liao, Paul G Voorhoeve, Willemijn G Leen, Nitash Zwaveling-Soonawala, V Krishna Chatterjee, Sjoerd A A Van den Berg, Frederik A Verburg, Marcel E Meima, Erica L T van den Akker, W Edward Visser

**Affiliations:** ^1^ Academic Center for Thyroid Diseases, Department of Internal Medicine, Erasmus Medical Center, Rotterdam, The Netherlands; ^2^ Department of Pediatrics, Canisius-Wilhelmina Hospital, Nijmegen, The Netherlands; ^3^ Department of Neurology, Canisius-Wilhelmina Hospital, Nijmegen, The Netherlands; ^4^ Department of Pediatric Endocrinology, Emma Children’s Hospital, Amsterdam UMC, University of Amsterdam, Amsterdam, The Netherlands; ^5^ Wellcome Trust-Medical Research Council Institute of Metabolic Science, University of Cambridge, Cambridge, UK; ^6^ Department of Clinical Chemistry, Erasmus Medical Center, Rotterdam, The Netherlands; ^7^ Department of Radiology and Nuclear Medicine, Erasmus Medical Center, Rotterdam, The Netherlands; ^8^ Division of Endocrinology, Department of Pediatrics, Erasmus MC-Sophia Children’s Hospital, University Medical Center, Rotterdam, The Netherlands

**Keywords:** resistance to thyroid hormone beta syndrome, thyroid hormone receptor beta, mosaicism, thyroid hormone analog, Triac

## Abstract

**Background:**

Patients with resistance to thyroid hormone β (RTHβ) show elevated thyroid hormone concentrations with non-suppressed thyroid-stimulating hormone (TSH) concentrations and large phenotypic variability. Triac therapy has been successfully applied in some patients. Mosaic mutations causing mild RTHβ have been reported three times so far.

**Patient:**

We present a case of severe RTHβ caused by a mosaic frameshift mutation in the thyroid hormone receptor β (p.R438Lfs445X). Methimazole and Triac combination therapy was commenced at the age of 8 years, resulting in a substantial decrease in free T4 concentrations and an increase in TSH concentrations (follow-up duration of >18 months). His extreme agitation, motor restlessness, weight, and some motor and communication skills improved. The mutation was fully unresponsive to stimulation with T3 in *in vitro* and *ex vivo* analyses.

**Conclusion:**

The p.R438Lfs445X mutation leads to a severe phenotype of RTHβ. Mosaicism might underlie a subset of patients with the clinical phenotype of RTHβ. Combined methimazole/Triac therapy had beneficial effects on several thyrotoxic features.

## Established facts

- Resistance to thyroid hormone β (RTHβ) is a condition caused by mutations in the thyroid hormone receptor beta. Disease features include elevated thyroid hormone concentrations with non-suppressed TSH concentrations, as well as variable features arising from a hypothyroid state in TRβ-expressing tissues and thyrotoxic features arising from TRα -expressing tissues. Neurocognitive and metabolic disturbances are typically mild.
- RTHβ caused by mosaic mutations is extremely rare, with only three cases reported so far.
- The thyroid hormone analog Triac is used as a therapeutic agent for RTHβ in some cases, although its effects are not well studied.

## Novel insights

- A novel 8-base pair duplication in *THRB* (c.1305_1312dup; (p.R438Lfs445X)) was identified in a mosaic pattern in a patient with severe RTHβ. This mutation resulted in a particularly severe developmental and thyrotoxic phenotype, which has not been previously observed in mosaic RTHβ.
- Combination therapy with methimazole and Triac proved necessary to achieve optimal thyroid hormone balance in this patient, as Triac alone caused paradoxical increases in TSH and free T4 concentrations, highlighting the complexity of treatment in mosaic RTHβ and the need for individualized therapeutic regimens.

## Introduction

Adequate thyroid hormone signaling is indispensable for many processes in the human body (1). Triiodothyronine (T3), the active thyroid hormone, exerts its effects through the nuclear thyroid hormone receptors (TR) α and β (1). Ligand binding results in the exchange of corepressors for coactivators binding to these receptors, with consequent alteration of the expression of thyroid hormone-sensitive genes, thereby regulating a large variety of downstream pathways (1). TRα is strongly expressed in the brain, heart, and bones, whereas TRβ is significantly expressed in the liver, kidneys, and hypothalamus, and pituitary (1). The TRβ isoform 2 (TRβ2) is specifically expressed in the pituitary, thereby playing a key role in negative feedback regulation (1).

Mutations in the genes encoding TRα and TRβ result in resistance to thyroid hormone (RTH) α and β, respectively (2, 3, 4). Patients with RTHα are characterized by hypothyroid symptoms in tissues predominantly expressing TRα (e.g. intellectual deficit, growth retardation, and bradycardia) (5). Patients with RTHβ typically show increased serum thyroid hormone concentrations with non-suppressed thyroid-stimulating hormone (TSH) concentrations, with TSH having higher bioactivity (6). Together, this indicates thyroid hormone resistance at the pituitary level, which may result in thyrotoxic symptoms in tissues predominantly expressing TRα (e.g. attention-deficit/hyperactivity disorder (ADHD), tachycardia, and atrial fibrillation) (7, 8). The symptomatology of RTHβ is strongly variable, ranging from no or mild/moderate symptoms in the majority of patients to rare cases with severe thyrotoxic symptoms and severe motor and intellectual disability (7, 9). The vast majority of patients carry a heterozygous mutation; the mutated allele restricts the function of the wild-type allele in a dominant-negative fashion. A limited number of patients with homozygous TRβ mutations have been reported, and many of those patients suffer from a severe phenotype (e.g. hypertrophic cardiomyopathy in childhood) (9, 10, 11, 12, 13, 14, 15). Remarkably, in 15% of patients with clinical signs of thyroid hormone resistance and no alternative diagnoses, no mutation in the gene encoding TRβ was identified (16).

In the majority of patients, no treatment is required due to mild symptomatology. Most patients who suffer from palpitations are well controlled by symptomatic beta-blocker therapy. The ADHD phenotype may improve upon L-T3 treatment in supraphysiologic doses (17). Treatment with the thyroid hormone analog Triac, either as sole treatment or in combination with the antithyroid drug propylthiouracil (PTU), has been applied in a limited number of patients, with substantial variety in efficacy (10, 15, 18, 19).

Here, we report an extreme case of RTHβ caused by a novel mosaic frameshift mutation, presenting with strongly elevated serum thyroid hormone concentrations, severe agitation, developmental delay, and metabolic consequences of thyrotoxicosis. We confirm the severity of the mutation using well-established *in vitro* and *ex vivo* models. We describe the effect of combined Triac/antithyroid drug treatment. These findings carry importance as they refine the phenotypic spectrum of RTHβ, highlight the relevance of mosaicism in the diagnostic work-up of patients suspected of RTHβ, and demonstrate the beneficial effects of combined antithyroid drug/Triac treatment in severe RTHβ cases.

## Case report

The proband is a Caucasian male, born at 35 + 3 weeks, with a birth weight of 2,402 grams (50th centile) (Supplementary information (see section on Supplementary materials given at the end of the article)). At 2 months of age, parents observed restlessness, excessive crying, and reflux as initial symptoms. Evaluations of his thyroid function tests showed markedly increased serum free T4 (>100 pmol/L), total T3 (8.2 nmol/L), and TSH (33 mU/L) concentrations. TSH receptor antibodies were negative. At 1.5 years of age, he developed seizures.

Analysis of *THRB* (encoding TRβ) was performed on DNA isolated from peripheral leukocytes at the age of 3 months. Results were interpreted as normal, consisting of homozygous wild-type TRβ.

After exclusion of other potential diagnoses and under persistent strong clinical suspicion of RTHβ, the results of the genetic analysis were revised 3 months later. Upon revision, a series of small additional peaks in exon 10 were identified, indicating a novel 8-base pair duplication at position 1,305 (c.1305_1312dup), leading to a frameshift and a premature stop codon (p.R438Lfs445X). Since the duplication was detected only in a small number of sequence reads, the diagnosis of mosaic RTHβ was made. Additional analysis of *THRB* on DNA isolated from buccal mucosal cells also revealed the c.1305_1312dup in an even smaller proportion of the sequence reads, thus confirming the diagnosis of mosaic RTHβ.

Throughout life, the proband had a low body weight-for-age, varying between −1 and −2 SD (Supplementary Fig. 1A), and subsequent low BMI (ranging from 11.6 to 13.8 kg/m^2^). In contrast, his height exceeded his parental target height range, suggesting that his bone maturation was abnormal (Supplementary Fig. 1B). Indeed, his bone age was remarkably advanced compared to his chronological age (approximately 4 years; Supplementary Fig. 1C). Moreover, he demonstrated a calcium-depleted skeleton.

Given his severe thyrotoxic phenotype and in the absence of alternative therapies, the proband was referred to our center for Triac treatment at 8 years of age. He demonstrated a phenotype of severe developmental delay and therapy-resistant generalized epilepsy. He was able to hold his head up but could not sit independently and was wheelchair-bound. He could not speak and only vocalized with repetitive sounds. He presented a multitude of symptoms of severe chronic thyrotoxicosis. He was in a state of severe continuous agitation and showed constant repetitive motion of his limbs and head. He showed a mean heart rate of 105 beats per minute (heart rate-for-age +2.18 SD, calculated with https://zscore.chboston.org/), frequent episodes of tachycardia, and premature atrial contractions on a 24 h Holter ECG reading. Transthoracic cardiac ultrasound showed a compressed right ventricle (due to pectus excavatum) and slightly increased velocity of flow over the tricuspid valve, with trivial tricuspid valve insufficiency but otherwise no structural cardiac abnormalities. Another clinical feature was his chronic diarrhea. He required continuous tube feeding to prevent nocturnal episodes of hypoglycemia.

Biochemical evaluation showed strongly elevated T3 (10.3 nmol/L, reference interval: 1.7–2.9 nmol/L) and free T4 (132.4 pmol/L, reference interval: 12.7–23.3 pmol/L) concentrations, with high TSH concentrations (5.88 mIU/L, reference interval: 0.60–5.22 mIU/L; Supplementary Table 1). Since TSH glycosylation assays require large volumes of blood, which was very difficult to obtain in this child, and the results of such assays would likely not alter clinical management, TSH glycosylation status (and thus TSH bioactivity) was not assessed. Ultrasound examinations showed a homogeneously increased thyroid volume (right and left lobes both 5.4 centimeters, total volume 41 mL) without focal abnormalities at 8.3 years of age.

(Bone) alkaline phosphatase was strongly increased, indicating a thyrotoxic state in his bones. SHBG, considered a marker for hepatic thyroid hormone action, was prominently elevated (Supplementary Table 1, Supplementary Fig. 2).

**Figure 1:**
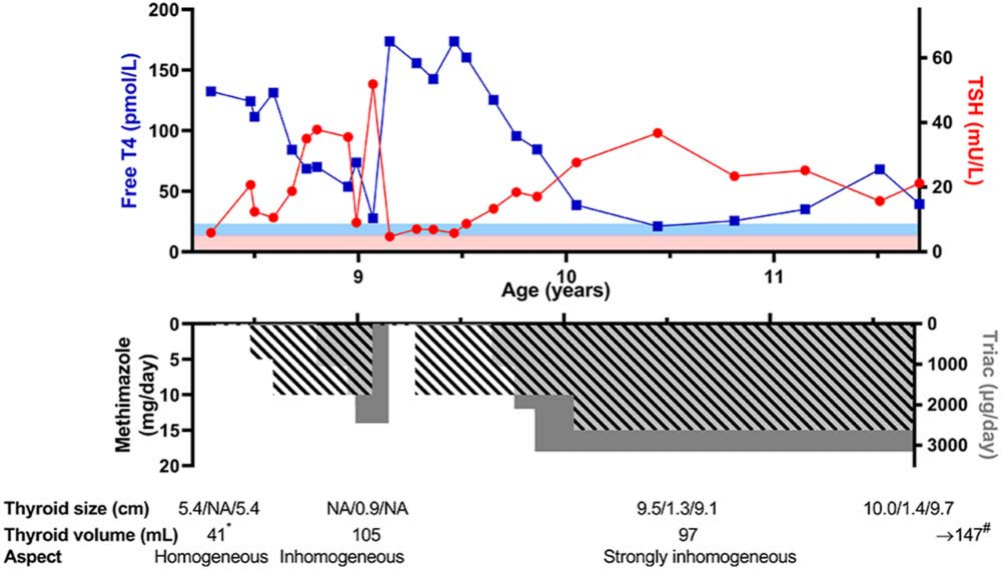
Clinical response to methimazole/Triac therapy. The light-blue bar indicates the age-specific reference interval for free T4; the light-red bar indicates the age-specific reference interval for TSH. The shaded bars indicate methimazole dose; the gray bars indicate Triac dose. Thyroid size is depicted as size of right lobe/size of isthmus/size of left lobe. Total thyroid volume was retrospectively assessed by an expert radiologist based on available ultrasound examinations, unless otherwise specified. *Thyroid volume was directly measured; the ultrasound technician deemed this an underestimation of actual thyroid volume. ^#^A measurement of thyroid volume based on a CT scan is presented here; this measurement was taken after follow-up (indicated by the arrow), while methimazole and Triac treatment was continued (in similar doses). T4, thyroxine; TSH, thyroid-stimulating hormone; NA, not available.

Before initiation of Triac treatment, an increasing dose of methimazole (5–10 mg (0.2–0.4 mg/Kg body weight)) was administered, aiming to prevent transiently increased thyrotoxic stress of Triac in addition to the elevated serum T3 concentrations (Fig. 1). Methimazole was chosen over PTU (despite PTU’s inhibiting effect on type 1 deiodinase, reducing conversion from T4 to T3) due to the significantly more benign hepatic safety profile of methimazole following clinical guidelines (20). Serum thyroid hormone concentrations decreased, and serum TSH concentrations increased. After 6 months of methimazole treatment, Triac therapy in an incrementing dose was added, aiming to reduce thyrotoxic stress by potent central suppression of the HPT axis (primarily via TSH suppression). Indeed, the introduction of Triac resulted in a further decrease in serum free T4 and total T3 (corrected for Triac interference) concentrations and clinical improvement. However, in contrast to the expected TSH suppression by Triac, serum TSH concentrations further increased and exceeded pre-treatment values. Consequently, the proband showed a goitrous thyroid (Fig. 1 and Supplementary Fig. 2). Therefore, methimazole was discontinued, aiming to restore HPT axis balance with Triac monotherapy, as previously described (18). However, free T4 concentrations drastically increased, even exceeding pre-treatment concentrations, while TSH concentrations normalized. We inferred from these observations that combined methimazole/Triac treatment was required to optimize thyroid hormone status in the different tissues of this patient, and thus weaned him off Triac monotherapy. Afterward, methimazole was restarted, and after 6 months Triac was reintroduced. On a final regimen of 15 milligrams methimazole and 3,150 μg Triac per day, serum free T4 concentrations had reached near-normal concentrations with relatively mildly increased serum TSH concentrations (Supplementary Table 1), not resulting in a severe goitrous phenotype (i.e. no obstruction).

Upon combined methimazole/Triac treatment, following the decrease in serum fT4 concentrations, several clinical sequelae rapidly improved. Parents reported that he was more alert, outgoing, and aware of his surroundings. He was much less agitated, and fewer repetitive motor movements were present. Moreover, treatment positively affected his metabolic state, as illustrated by positive body weight-for-age development and a reduced need for nutritional support via his feeding tube to prevent nocturnal hypoglycemia. His bone age advancement decreased upon treatment (Supplementary Fig. 1C). No adverse effects, other than the goiter, were observed during >18 months of follow-up on stable doses of methimazole/Triac.

## Materials and methods

Clinical examinations were carried out in the context of routine care and were retrospectively described. This study was conducted in agreement with the Medical Research Involving Human Subjects Act and in accordance with the Declaration of Helsinki. A waiver was provided by the Ethics Committee of the Erasmus Medical Center. Written informed consent has been obtained from the patient (or the patient’s guardian) for publication of the case report and accompanying images.

For complete Materials and Methods, see Supplementary information.

## Results

**Figure 2:**
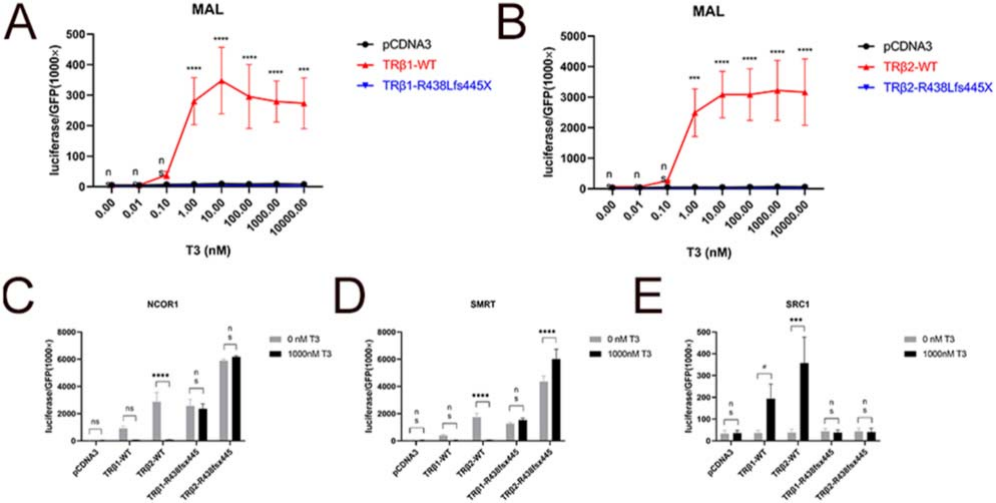
Receptor activity on reporter construct MAL upon stimulation with increasing concentrations of T3. (A) TRβ1 isoform; (B) TRβ2 isoform. Recruitment/dissociation upon stimulation with 1,000 nM T3 (in both TRβ1 and -2 isoform); (C) NCOR1; (D) SMRT; (E) SRC1. Two-way ANOVA followed by Bonferroni posttests (A and B) and multiple *t*-test followed by Holm-Sidak posttests (C, D, E) were applied to assess statistically significant differences between tested conditions (ns, not significant; ^#^*P* < 0.1 (borderline significant); ***P < 0.0005; ****P < 0.0001). MAL, malic enzyme; GFP, green fluorescent protein; WT, wild-type; NCOR1, nuclear receptor co-repressor 1; SMRT, silencing mediator for retinoid or thyroid-hormone receptors; SRC1, steroid receptor coactivator 1.

In contrast to WT TRβ1 and TRβ2, stimulation of the R438Lfs445X mutant with increasing concentrations of T3 did not result in increased receptor activity *in vitro* (Fig. 2A and B). Release of co-repressors NCOR1 and SMRT and binding of co-activator SRC1 upon T3 stimulation were nullified in both TRβ1 and TRβ2 contexts (Fig. 2C, D, E).

Analysis of exon 10 of *THRB* on gDNA isolated from the proband’s skin fibroblasts demonstrated double peaks starting at the same position as observed in gDNA isolated from buccal mucosal cells (Supplementary Fig. 3A), making these fibroblasts a suitable model to study thyroid hormone responsiveness (21). In contrast to fibroblasts derived from a healthy control, patient-derived fibroblasts did not show a significant increase in *KLF9* expression upon stimulation with 10 nM T3 (Supplementary Fig. 3B), confirming the severe thyroid hormone-resistant phenotype.

## Discussion

Here, we present a case of severe RTHβ caused by a mosaic mutation in *THRB*. Moreover, we demonstrate beneficial effects of combined anti-thyroid drug/Triac therapy.

This case highlights several important messages for clinical care of patients with RTHβ. First, in contrast to the majority of patients with RTHβ who are asymptomatic or experience mild symptoms, this patient showed gross abnormalities and symptoms due to altered thyroid hormone action in several organ systems. Such cases of severe heterozygous RTHβ, as illustrated by extremely elevated serum TSH and free T4 concentrations, have only been reported scarcely, with a subset of these patients also showing severe intellectual and motor disability (12, 13, 14, 15, 19). Interestingly, the mutations of these patients share high similarity, all being frameshifts starting in amino acid residues 435–453 and resulting in premature truncation of the C-terminus of the protein. It has been previously suggested that this region has particular importance in interaction with the corepressor protein SMRT (19). The presented data do not support a pathogenic mechanism driven by altered SMRT interaction *per se*, and additional studies are needed to unravel mutual pathogenic factors in such severely affected patients. The severity of the phenotype and its response to treatment with antithyroid drugs and Triac also resemble the clinical presentation of homozygous RTHβ patients (10). Thus, treatment with antithyroid drugs and Triac might be considered in all severely affected RTHβ patients (irrespective of homo- or heterozygosity of the mutation). We deem it more likely that the underlying mutation drives the severe phenotype of the presented case rather than the mosaic expression of the mutation, since other reported mosaic RTHβ patients (albeit a very limited number) do not show a more severe phenotype compared to non-mosaic patients with similar mutations (16, 22, 23).

Second, this case re-emphasizes the possibility of mosaicism in the diagnostic work-up of RTHβ. Critical revision and/or sequencing of *THRB* on gDNA isolated from other tissues might prevent missed RTHβ diagnoses. Indeed, although an extra-rare entity within a rare disease, three other RTHβ cases caused by mosaicism have been reported in the literature (16, 22, 23). Interestingly, SHBG, a marker for hepatic thyroid hormone action, was strongly increased, which indicates that his liver is not resistant to thyroid hormone (and thus likely expresses WT TRβ). This observation is in strong contrast with another non-mosaic RTHβ patient with a severe phenotype who showed normal SHBG concentrations (12), and demonstrates the relevance of understanding the mutational pattern of mosaic patients. To our knowledge, this is the first report of RTHβ mosaicism resulting in differential clinical effects in tissues predominantly expressing TRβ.

Third, we observed that combination therapy with the anti-thyroid drug methimazole and the T3 analog Triac can alleviate the thyrotoxic phenotype in severe RTHβ, whereas monotherapy with either of these drugs did not. This is in contrast to most previous observations of Triac monotherapy improving clinical sequelae in patients with RTHβ (15, 18). However, a severely thyrotoxic RTHβ patient was treated with a combination of PTU and Triac, resulting in a similar decrease in serum thyroid hormone and TSH concentrations (19). It is unclear whether initiation of combined methimazole/Triac treatment earlier in life would have modified neurodevelopmental outcomes in this particular patient. Notably, serum fT4 and TSH concentrations showed different responses following the (re)initiation of methimazole/Triac treatment. Difficulties in achieving intake and uptake of medication (due to intercurrent illness, commonly observed in this vulnerable patient) may underlie these differential effects and also affect timing of treatment (reintroduction of Triac was delayed because of intercurrent illness). The delicate balance of treatment compliance is exemplified by a brief episode with gastrointestinal problems at 9 years of age, directly resulting in spiking serum fT4 concentrations and subsequent diminishing serum TSH concentrations (Fig. 1). In addition, it remains unclear how the mosaic state of this case influences the efficacy of the different treatment regimens applied. Additional studies on the mechanism of action of Triac in RTHβ are warranted.

Our observations illustrating the relevance of mosaicism in RTHβ diagnosis and the beneficial effects of combined methimazole/Triac therapy in severe RTHβ may inform clinical care for patients with RTHβ.

## Supplementary materials



## Declaration of interest

FS van Geest serves as a paid consultant for Egetis Therapeutics (the manufacturer of Triac). Egetis Therapeutics had no influence on the conduct or analysis of this study. All other authors declare no competing interests or funding.

## Funding

The center in Cambridge (UK) is supported by the Wellcome Trusthttps://doi.org/10.13039/100010269 (Investigator Award 210755/Z/18/Z to VKC) and the National Institute of Health Research Cambridge Biomedical Research Centre. The Erasmus Medical Center (Rotterdam, Netherlands), which employs FSvG, WL, SAAvdB, MEM, ELTvdA, and WEV , receives royalties from Egetis Therapeutics (the manufacturer of Triac). None of the authors benefits personally from these royalties. The Academic Center for Thyroid Diseases, Erasmus Medical Center, Rotterdam, is part of the European Reference Network on rare endocrine conditions (Endo-ERN).

## Patient consent

Written informed consent was obtained from the patient's legal guardian for publication of the submitted article and accompanying images.

## Author contribution statement

FSvG, PGV, WGL, NZ-S, FAV, ELTvdA, and WEV performed clinical or radiological evaluations. FSvG, ELTvdA, and WEV treated the described patient. FSvG, WL, and MEM performed the experiments and analyses. VKC provided expert consultation. SAAvdB performed biochemical analyses. FSvG wrote the manuscript. All other authors reviewed the manuscript.
