# Supplementary material for: Extreme resistance to thyroid hormone caused by a novel mosaic thyroid hormone receptor beta mutation
Source: Eur Thyroid J. 2025 Nov 11;14(6):e250092. doi: 10.1530/ETJ-25-0092 (PMC12608061; doi:10.1530/ETJ-25-0092)
Supplement: Supplementary file 1 [file supplementary_materials.pdf]

Supplementary information to

Extreme resistance to thyroid hormone caused by a novel mosaic thyroid hormone receptor beta mutation: a case report

Ferdy S. van Geest, Wenjun Liao, Paul G. Voorhoeve, Willemijn G. Leen, Nitash Zwaveling-Soonawala, V. Krishna Chatterjee, Sjoerd A.A. van den Berg, Frederik A. Verburg, Marcel E. Meima, Erica L.T. van den Akker, W. Edward Visser

Additional clinical information

The proband is a Caucasian male, born to healthy non-consanguineous parents. He had a birth weight at the 50th percentile (1). His birth was complicated by umbilical cord entanglement and meconium stained amniotic fluid and he required antibiotics for 7 days postpartum. Auditory screening at 2 weeks of age showed impaired hearing. Consequent evaluations at an auditory center found a perceptive hearing loss with a hearing threshold of 60 dB, and he has utilized hearing devices since the age of 5 months.

His developmental age was delayed compared to his chronological age, resembling that of a healthy child 5 to 6 months at the age of 10 months (biological age after correction for prematurity: 9 months; Kent Infant Development Scale – Dutch version (KID-N, (2, 3))). Development upon aging was limited, as he showed a developmental age of 7 months at the age of 2 years and 5 months and a development age of 6 to 11 months at 8 years of age.

Magnetic resonance imaging (MRI) at 3 months of age showed diffuse low cerebral white matter volume and a thin corpus callosum, but no hypomyelination and otherwise normal cerebral structures and no morphological abnormalities of the hypothalamus and anterior and posterior pituitary. Repetitive MRI at 1 year and 3 months of age showed no hypomyelination, but did show microcephaly, a slight loss of white matter volume and a normal pituitary. Cardiac screening using transthoracic ultrasound at age 3.5 years showed no structural abnormalities.

Given the presence of clinical features of thyrotoxicosis at 3 months of age, beta-blocker therapy was initiated. Subsequently, his heart rate decreased and other thyrotoxic features became less outspoken. The proband was treated with beta-blocker therapy for multiple years. It was discontinued due to potential contribution to hypoglycaemias at that time and slight preference by parents. During combined methimazole/Triac treatment, he did not receive beta-blocker therapy.

Despite nutritional support by tube feeding tube since he was 2 years old, the proband regularly suffered from hypoglycaemia. A fasting test showed hypoglycaemia after only 14 hours. During his

life, the proband was recurrently admitted for complications of his feeding tube (dislocations, infections with consequent hypoglycaemia). Moreover, he suffered from recurrent infections in the ear-nose-throat region, for which he received regular prophylactic antibiotic treatment. IgG-subclass analysis at the age of 8 years identified no relevant abnormalities (IgG2 0.76 g/L, reference interval 0.85-4.10 g/L; IgG4 0.01 g/L, reference interval <1.89 g/L). Excessive sialorrhoea was adequately treated with botox injections.

Methods

Biochemical analyses

Serum fT4 concentration measurements were recalibrated to the reference method (4). Serum TSH concentration measurements were recalibrated according to the recommendations of The International Federation of Clinical Chemistry and Laboratory Medicine (IFCC) (5). All other biochemical analyses were performed according to manufacturer's instructions, as previously described (6, 7).

DNA constructs and mutagenesis

The construction of pcDNA3 FLAG-TRβ1 and (pituitary-specific) TRβ2 expression vectors containing full-length human TRβ1 and TRβ2 with 5' FLAG tagged was performed as described previously (8). The c.1305_1312dup variant, translating to p.R438Lfs445X, was introduced in all vectors using the QuickChange II Mutagenesis kit (Agilent Technologies, Amstelveen, Netherlands) according to the manufacturer's protocol. The introduced mutation was confirmed by Sanger sequencing and introduction of other mutations was excluded.

Cell culture and transfection

JEG-3 cells (ECACC Cat. no. 92120308, RRID:CVCL_0363; Sigma-Aldrich, Munich, Germany) were cultured and transfected as previously described (8). For transcriptional activity assays, WT or mutant receptors were coexpressed with luciferase reporter constructs containing direct repeat TH response

elements (DR4-TRE) as well as pMaxGFP as a transfection control. Two-hybrid assays were performed as previously described, using plasmids containing the interactive domain of the specific cofactors (9). Briefly, 10 ng of VP16-TRβ plasmid was cotransfected with 10 ng GAL4-NCOR1, GAL4-SMART or GAL4-SRC1 and 120 ng of UAS-tkLuc, as well as 60 ng pMaxGFP as a transfection control. After transfection for 24 hours, cells were starved from thyroid hormone for 24 hours and subsequently stimulated with indicated concentrations T3 (Cat. no. T2877; Sigma-Aldrich) in DMEM/F12 medium supplemented with 0.1% bovine serum albumin for 24 hours.

Fibroblasts derived from the proband and a healthy control were cultured as described previously (10). At >95% confluency, cells were starved from thyroid hormone for 24 hours and subsequently stimulated with indicated concentrations T3 (Cat. no. T2877; Sigma-Aldrich) in DMEM/F12 medium supplemented with 0.1% bovine serum albumin for 24 hours.

Luciferase assays

Luciferase activity was measured as previously described (8). The results are shown as the mean ± SEM of at least three independent experiments performed in triplicate.

Genetic analyses

Genomic DNA was extracted from fibroblasts using a DNeasy® Blood & Tissue Kit (Qiagen) according to the manufacturer's instructions. Sanger sequencing was performed using routine methods. Primers are available upon request. The National Center for Biotechnology Information reference sequence NM_000461.4 was used.

Quantitative polymerase chain reaction

Total RNA was extracted from fibroblasts in 6-well plates using TRI Reagent (Sigma-Aldrich) according to the manufacturer’s protocol, and cDNA was produced using the Transcriptor High Fidelity cDNA Synthesis Kit (Roche Diagnostics) according to the manufacturer`s protocol. Quantitative polymerase

chain reaction was performed as previously described, using probe-based assays for the detection of *KLF9* and *GAPDH* (11).

Ethical considerations

Skin fibroblasts were collected for diagnostic purposes by caregiving physicians, with written informed consent of the parents. Clinical examinations were carried out in the context of routine care and were retrospectively described. This study was conducted in agreement with the Medical Research Involving Human Subjects Act and in accordance with the Declaration of Helsinki. A waiver was provided by the Ethics Committee of the Erasmus Medical Center.

Statistical analyses

All statistical analyses were carried out using GraphPad Prism version 9 (GraphPad Software, San Diego, California, USA). The applied statistical tests and levels of significance are indicated in the legend of the corresponding figure.

Supplementary table 1: Biochemical evaluations

Biochemical parameter	Baseline	Treated	Baseline relative to R.I.	Treated relative to R.I.	Reference interval
**Thyroid**					
TSH	5.88	21.2			0.6-5.2 mU/L
Free T4	132.4	39.4	5.7 x URL		12.72-23.28 pmol/L*
Total T4	373	148			80-141 nmol/L
Total T3	10.29	7.02			1.7-2.9 nmol/L#
Reverse T3	1.88	0.79			0.2-0.5 nmol/L
**Liver**					
ASAT	42	31			<51 U/L
ALAT	72	48			<39 U/L
Lactate dehydrogenase	238	210			<425 U/L
Gamma-GT	80	65			<17 U/L
SHBG	373	177.8			40-140 nmol/L
Triglyceride	0.98				0.3-1.1 mmol/L
Total cholesterol	3.0	2.6			2.8-5.4 mmol/L
LDL cholesterol	1.80	1.18			1.2-3.4 mmol/L
HDL cholesterol	1.16				0.8-1.9 mmol/L
**Kidneys**					
Creatinin	39	42			31-68 µmol/L
Urea	7.1	7.7			3.3-5.6 mmol/L
**Bones**					
Alkaline phosphatase	505	487			<425 U/L
Bone alkaline phosphatase	205.0	90.0	14.3 x URL	6.3 x URL	<14.3 µg/L
P.I.N.P.	508				200-900 ng/mL
**Haematology**					
Haemoglobin	9.1				6.6-8.4 mmol/L
Haematocrit	0.43				0.32-0.40 L/L
MCV	79				74-88 fl
RDW	13.7				12.0-14.0 %
Trombocytes	267				199-369 x10^9/L
Leukocytes	7.7				4.3-11.4 x10^9/L
**Other**					
Ferritin	25	52			30-240 µg/L
Creatine kinase	89	151			<230 U/L

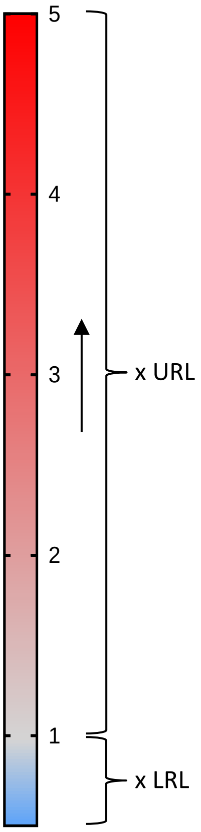

Biochemical analyses before initiation of treatment at age 8.3 years and on combined methimazole/Triac treatment at age 11.6 years. * Reference range changed to 13.1-24.0 pmol/L for analyses on treatment. #Total T3 concentrations on treatment were corrected for Triac interference as previously described (7). Abbreviations: R.I., reference interval; URL, upper reference limit; LRL, lower reference limit; TSH, thyroid stimulating hormone; T4, thyroxine; T3, triiodothyronine; ASAT, aspartate aminotransferase; ALAT, alanine aminotransferase; GT, glutamyltransferase; SHBG, sex hormone binding globulin; LDL, low density lipoprotein; HDL, high density lipoprotein; P.I.N.P, procollagen type 1 N-propeptide; MCV, mean corpuscular volume; RDW, relative distribution width.

Supplementary figure 1: Development of anthropometric outcomes over time

A

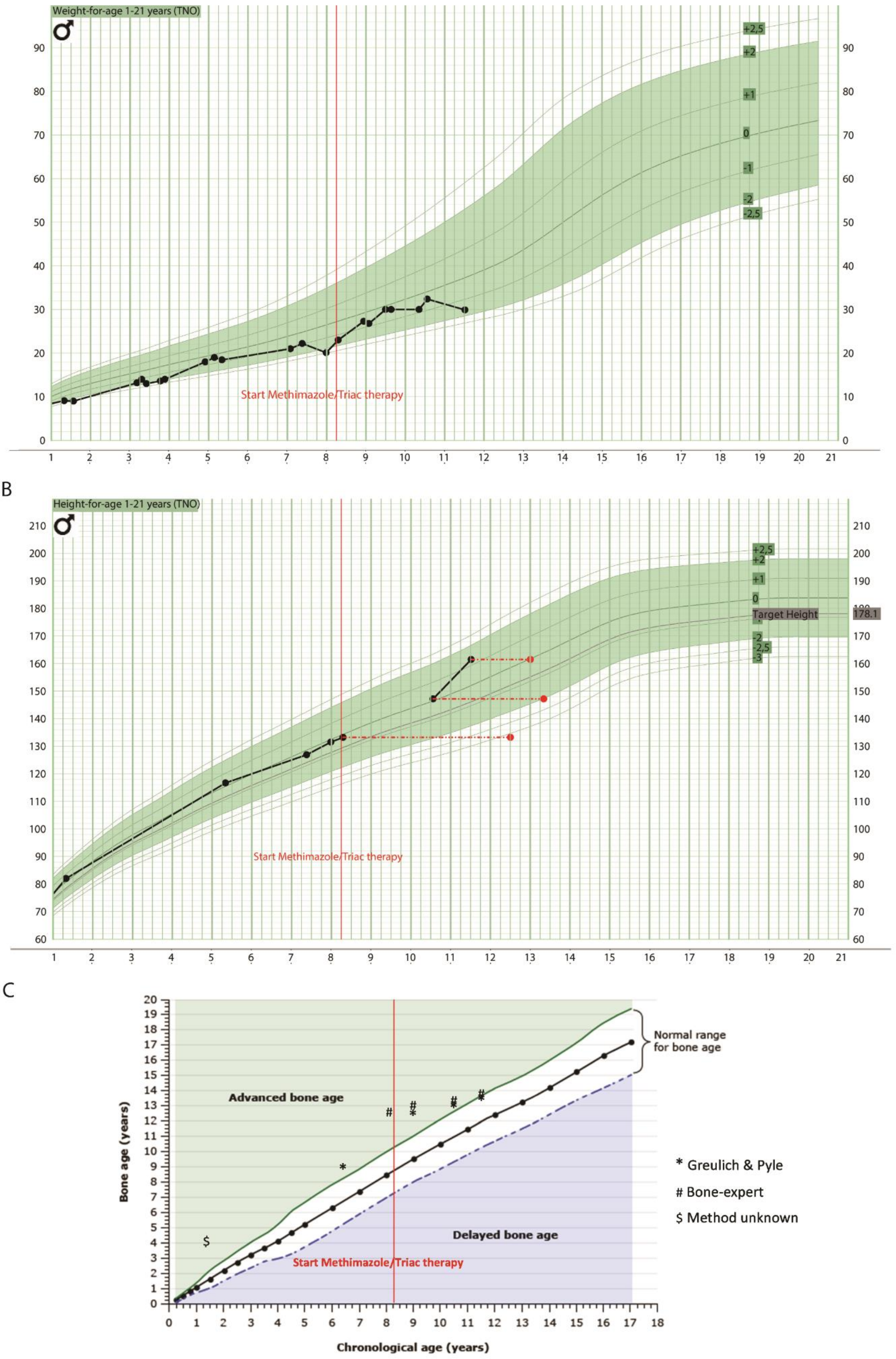

Supplementary figure 1: Development of weight-for-age (A), height-for-age (B), and bone age-for-age (C) (12, 13). Red dots in B illustrate the bone age at the age of measurement (black dot). The proband was prepubertal at all evaluations. The original reference range graph for bone-age-for-age (used in C) is derived from uptodate.com (14).

Supplementary figure 2: Thyroid hormone action in different tissues in different patients with RTHβ

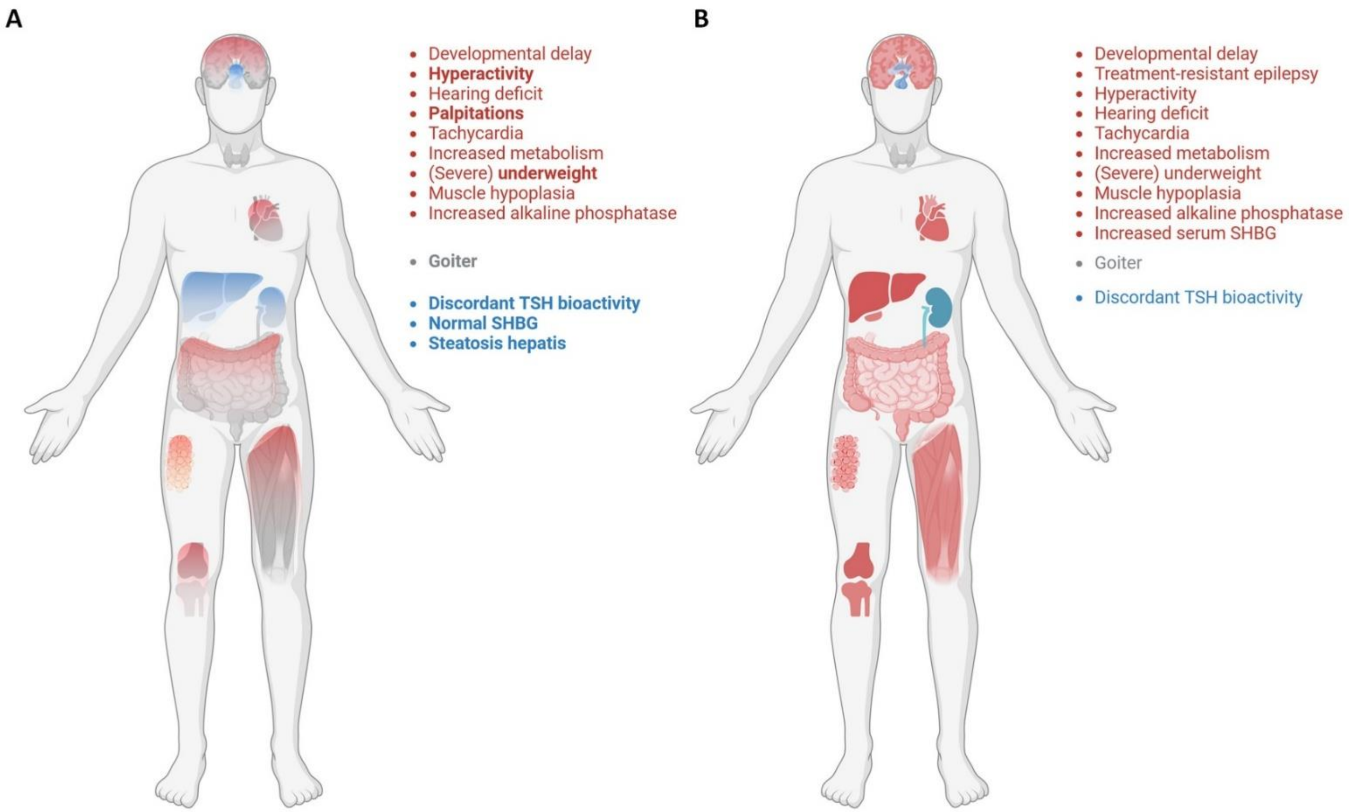

Supplementary figure 2: Clinical status of thyroid hormone action in different thyroid hormone target tissues in patients with RTHβ, ranging from mild (15, 16) to severe (indicated by color gradients), with symptoms commonly observed in patients with a milder phenotype in bold (A) (17-20), and the proband (B). Red indicates tissues with supraphysiological thyroid hormone action; grey indicates tissues with physiological thyroid hormone action; blue indicates tissues with infraphysiological thyroid hormone action. Abbreviations: TR, thyroid hormone receptor; TSH, thyroid stimulating hormone; SHBG, sex hormone-binding globulin. Figure created with http://www.Biorender.com.

Supplementary figure 3: Analysis of patient-derived fibroblasts

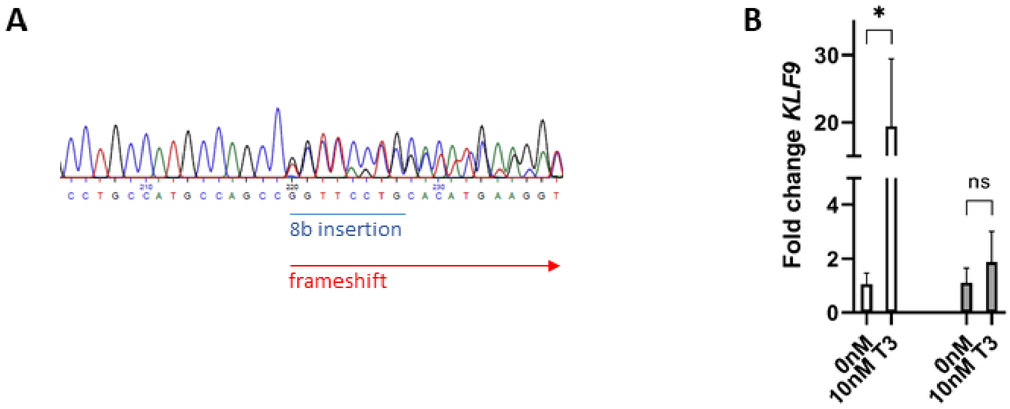

Supplementary figure 3: Results of genetic analysis of the affected part of exon 10 of *THRB* on gDNA isolated from patient-derived fibroblasts (A). The blue line indicates the mutation on DNA level, and the red line indicates its downstream consequences on DNA level. Induction of expression of thyroid hormone sensitive gene *KLF9* by stimulation with 10 nM T3 in fibroblasts derived from a healthy control (white bars) and the proband (grey bars) (B). Unpaired T tests were applied to assess for statistically significant differences between the tested conditions (ns, not significant; *, $p<0.05$).

References

1. Villar J, Cheikh Ismail L, Victora CG, Ohuma EO, Bertino E, Altman DG, et al. International standards for newborn weight, length, and head circumference by gestational age and sex: the Newborn Cross-Sectional Study of the INTERGROWTH-21st Project. Lancet. 2014;384(9946):857-68.
2. Katoff L, Reuter J, Dunn V. The Kent Infant Developmental scale manual. Kent, OH: Kent State University; 1978.
3. Schneider MJ, Loots GMP, Reuter J. Kent Infant Development Scale. Nederlandse bewerking. Handleiding. Lisse: Swets en Zeitlinger; 1990.
4. De Grande LAC, Van Uytfanghe K, Reynders D, Das B, Faix JD, MacKenzie F, et al. Standardization of Free Thyroxine Measurements Allows the Adoption of a More Uniform Reference Interval. Clin Chem. 2017;63(10):1642-52.
5. Thienpont LM, Van Uytfanghe K, De Grande LAC, Reynders D, Das B, Faix JD, et al. Harmonization of Serum Thyroid-Stimulating Hormone Measurements Paves the Way for the Adoption of a More Uniform Reference Interval. Clin Chem. 2017;63(7):1248-60.
6. Groeneweg S, Peeters RP, Moran C, Stoupa A, Auriol F, Tonduti D, et al. Effectiveness and safety of the tri-iodothyronine analogue Triac in children and adults with MCT8 deficiency: an international, single-arm, open-label, phase 2 trial. Lancet Diabetes Endocrinol. 2019;7(9):695-706.
7. van Geest FS, Groeneweg S, van den Akker ELT, Bacos I, Barca D, van den Berg SAA, et al. Long-Term Efficacy of T3 Analogue Triac in Children and Adults With MCT8 Deficiency: A Real-Life Retrospective Cohort Study. J Clin Endocrinol Metab. 2022;107(3):e1136-e47.
8. Wejaphikul K, Groeneweg S, Dejkhamron P, Unachak K, Visser WE, Chatterjee VK, et al. Role of Leucine 341 in Thyroid Hormone Receptor Beta Revealed by a Novel Mutation Causing Thyroid Hormone Resistance. Thyroid. 2018.
9. Wejaphikul K, van Gucht ALM, Groeneweg S, Visser WE, Visser TJ, Peeters RP, Meima ME. The In Vitro Functional Impairment of Thyroid Hormone Receptor Alpha 1 Isoform Mutants Is Mainly Dictated by Reduced Ligand Sensitivity. Thyroid. 2019;29(12):1834-42.
10. Groeneweg S, van den Berge A, Meima ME, Peeters RP, Visser TJ, Visser WE. Effects of Chemical Chaperones on Thyroid Hormone Transport by MCT8 Mutants in Patient-Derived Fibroblasts. Endocrinology. 2018;159(3):1290-302.
11. Kersseboom S, Horn S, Visser WE, Chen J, Friesema EC, Vaurs-Barriere C, et al. In vitro and mouse studies supporting therapeutic utility of triiodothyroacetic acid in MCT8 deficiency. Mol Endocrinol. 2014;28(12):1961-70.
12. Greulich WW, Pyle SI. Radiographic Atlas of Skeletal Development of the Hand and Wrist, 2nd Edition: Stanford, CA: Stanford University Press and London, UK: Oxford University Press; 1959.
13. Gaskin CM, Kahn SL, Bertozzi JC, Bunch PM. Skeletal development of the hand and wrist: Oxford University Press, New York; 2011.
14. https://www.uptodate.com/contents/image?imageKey=PEDS%2F94179~PEDS%2F94180 [
15. Pappa T, Refetoff S. Resistance to Thyroid Hormone Beta: A Focused Review. Front Endocrinol (Lausanne). 2021;12:656551.
16. Beck-Peccoz P, Chatterjee VK. The variable clinical phenotype in thyroid hormone resistance syndrome. Thyroid. 1994;4(2):225-32.
17. Phillips SA, Rotman-Pikielny P, Lazar J, Ando S, Hauser P, Skarulis MC, et al. Extreme thyroid hormone resistance in a patient with a novel truncated TR mutant. J Clin Endocrinol Metab. 2001;86(11):5142-7.
18. Maruo Y, Mori A, Morioka Y, Sawai C, Mimura Y, Matui K, Takeuchi Y. Successful every-other-day liothyronine therapy for severe resistance to thyroid hormone beta with a novel THRB mutation; case report. BMC Endocr Disord. 2016;16:1.
19. Behr M, Ramsden DB, Loos U. Deoxyribonucleic acid binding and transcriptional silencing by a truncated c-erbA beta 1 thyroid hormone receptor identified in a severely retarded patient with resistance to thyroid hormone. J Clin Endocrinol Metab. 1997;82(4):1081-7.

20. Gurgel MH, Montenegro Junior RM, Magalhaes RA, Lima GE, Montenegro RM, Magalhaes PK, Maciel LM. E449X mutation in the thyroid hormone receptor beta associated with autoimmune thyroid disease and severe neuropsychomotor involvement. Arq Bras Endocrinol Metabol. 2008;52(8):1205-10.
